# Supplementary material for: De Novo Assembly and Characterization of the Transcriptome of an Omnivorous Camel Cricket (Tachycines meditationis)
Source: Int J Mol Sci. 2023 Feb 16;24(4):4005. doi: 10.3390/ijms24044005 (PMC9966759; doi:10.3390/ijms24044005)
Supplement: Supplementary file 1 [file ijms-24-04005-s001.zip › ijms-2170599 supplementary figures final.pdf]

Supplementary Figures

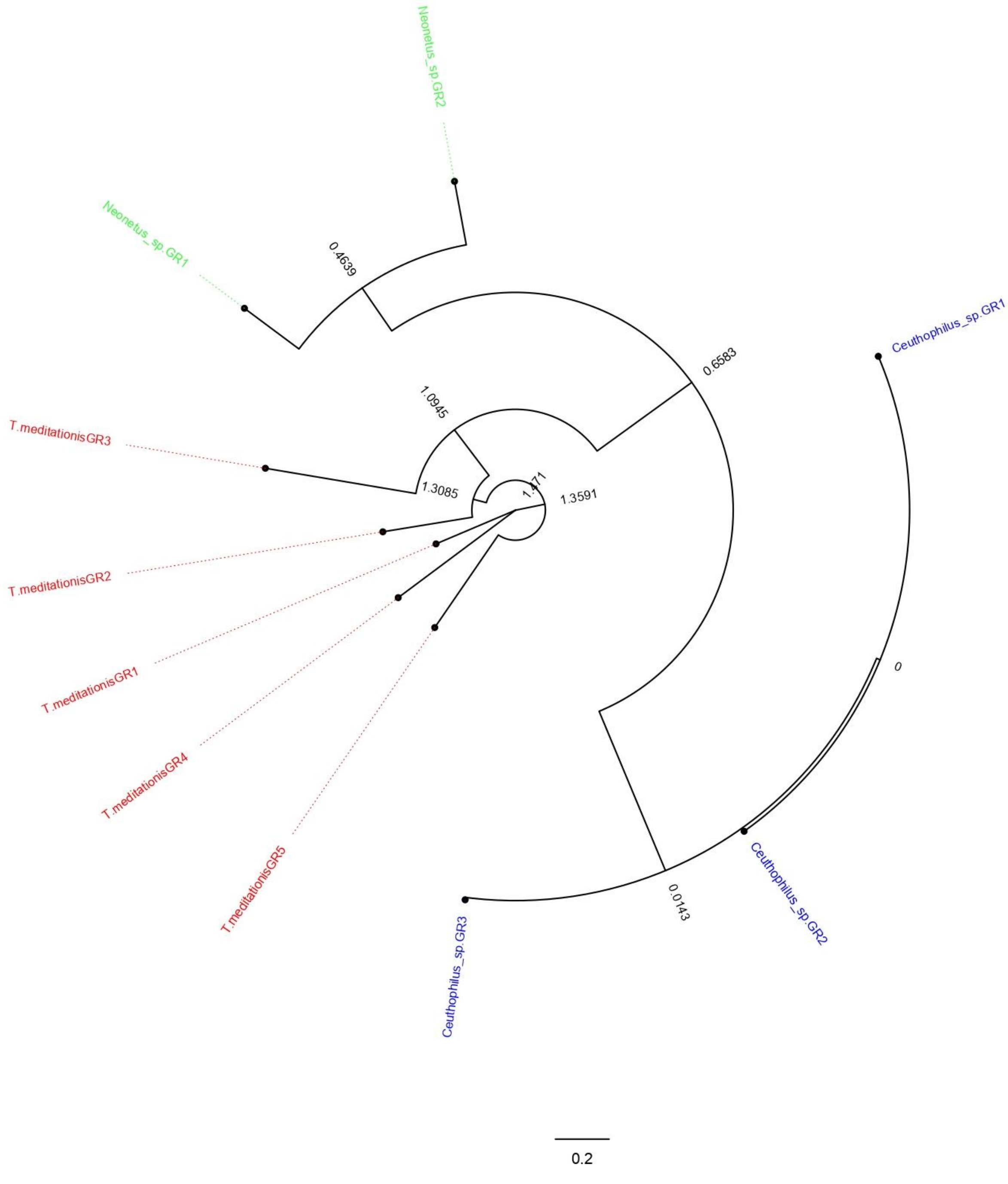

Figure S1. The ML phylogenetic tree of GR genes.

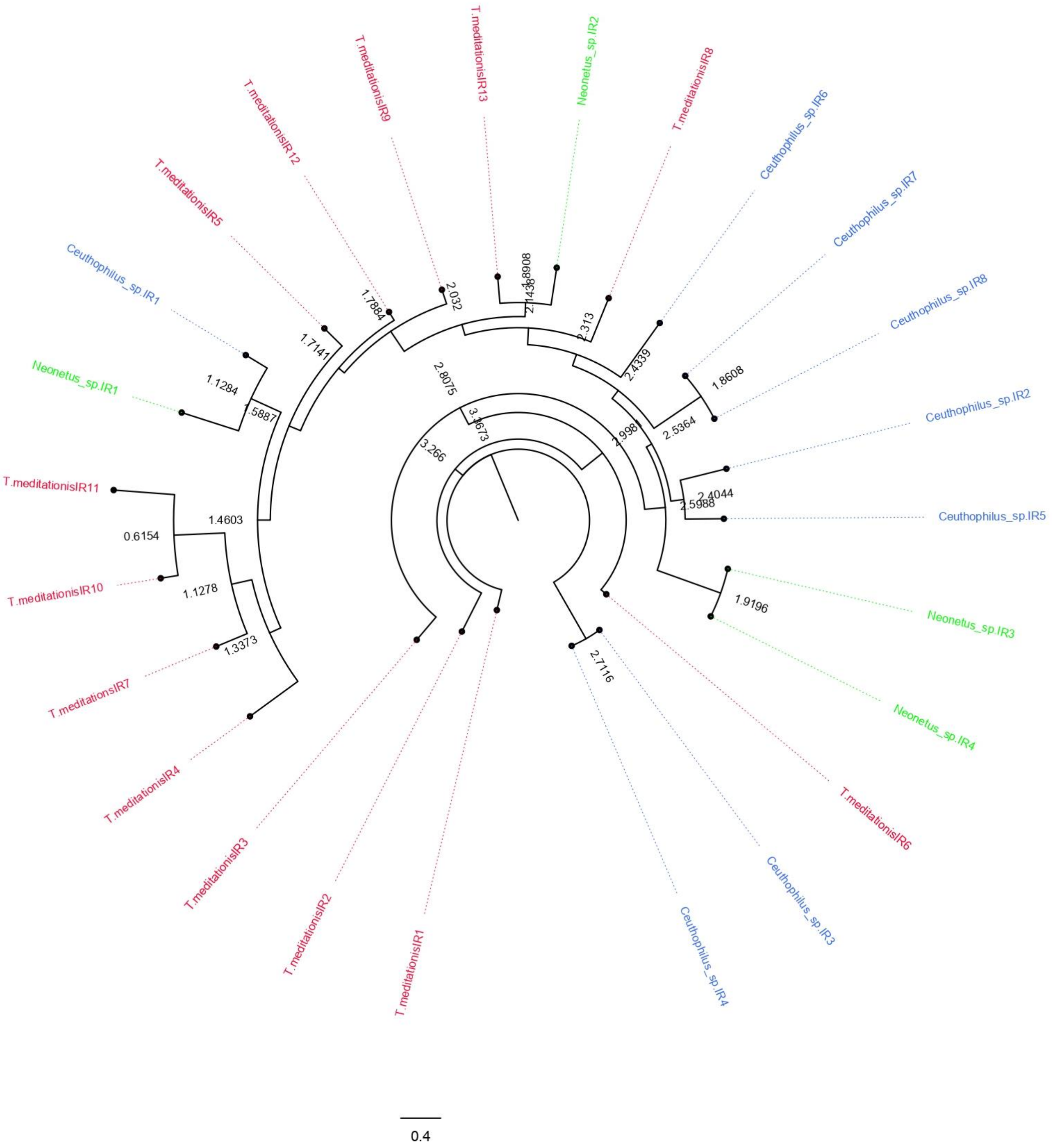

Figure S2. The ML phylogenetic tree of IR genes.

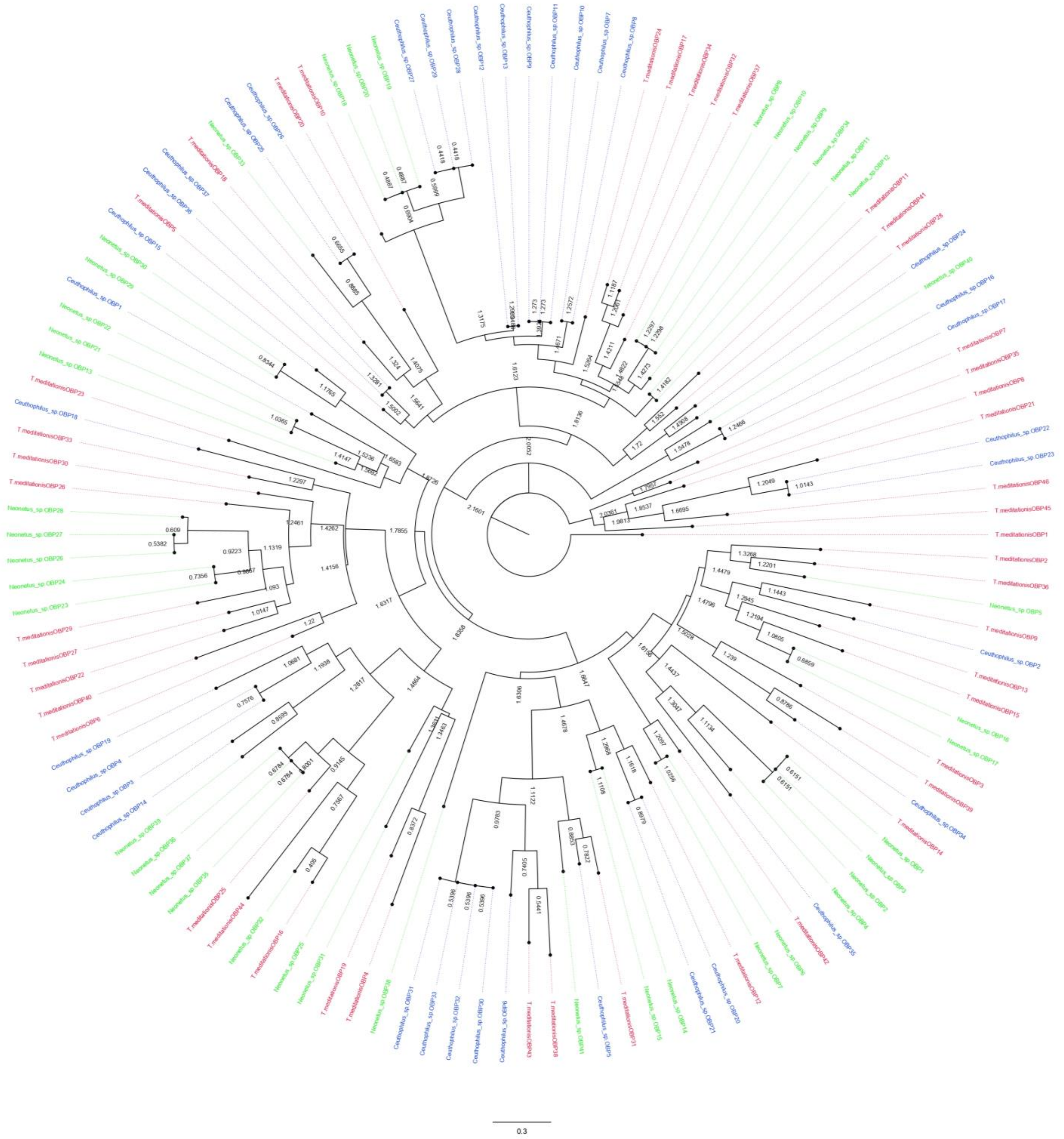

Figure S3. The ML phylogenetic tree of OBP genes.

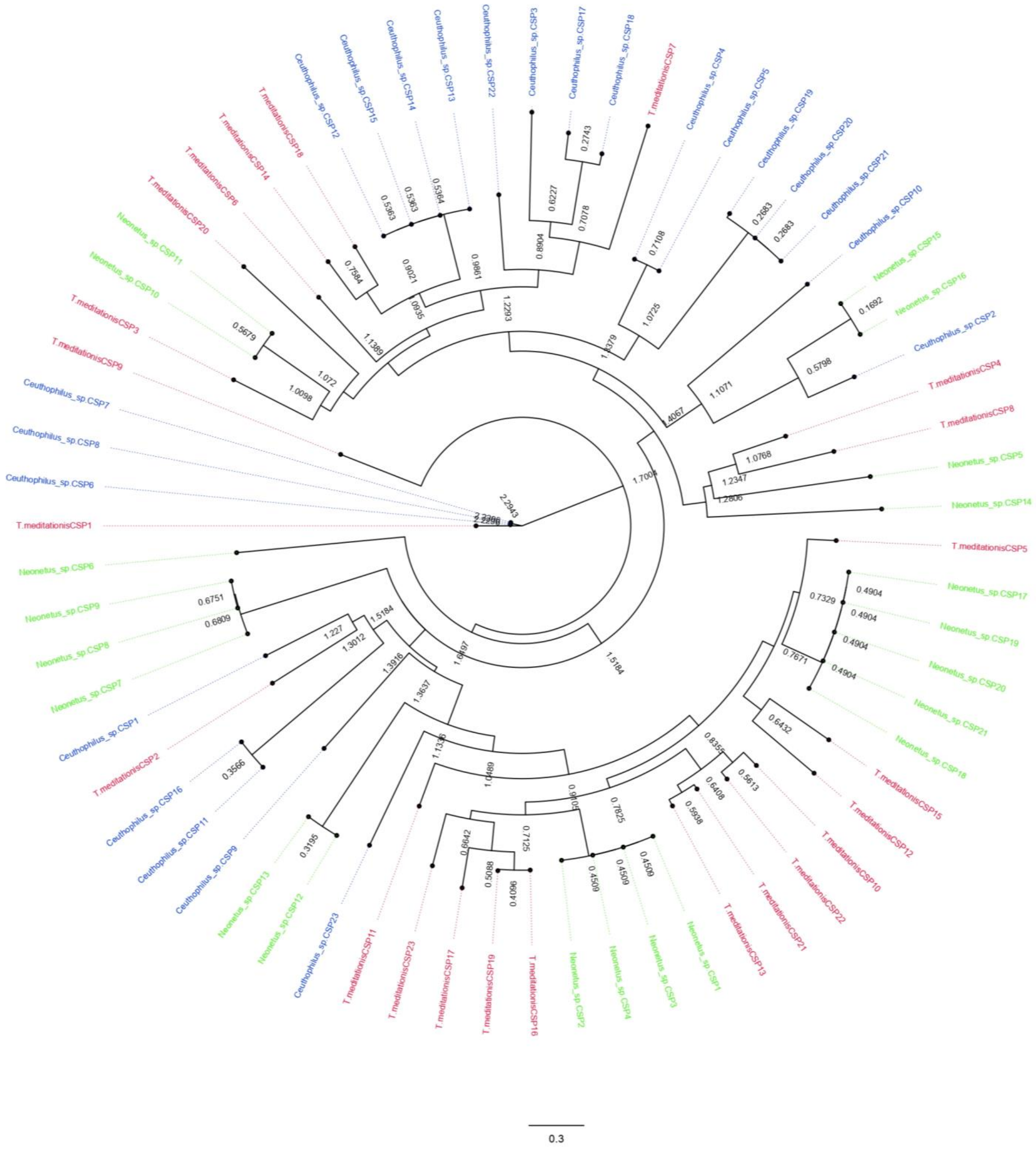

Figure S4. The ML phylogenetic tree of CSP genes.

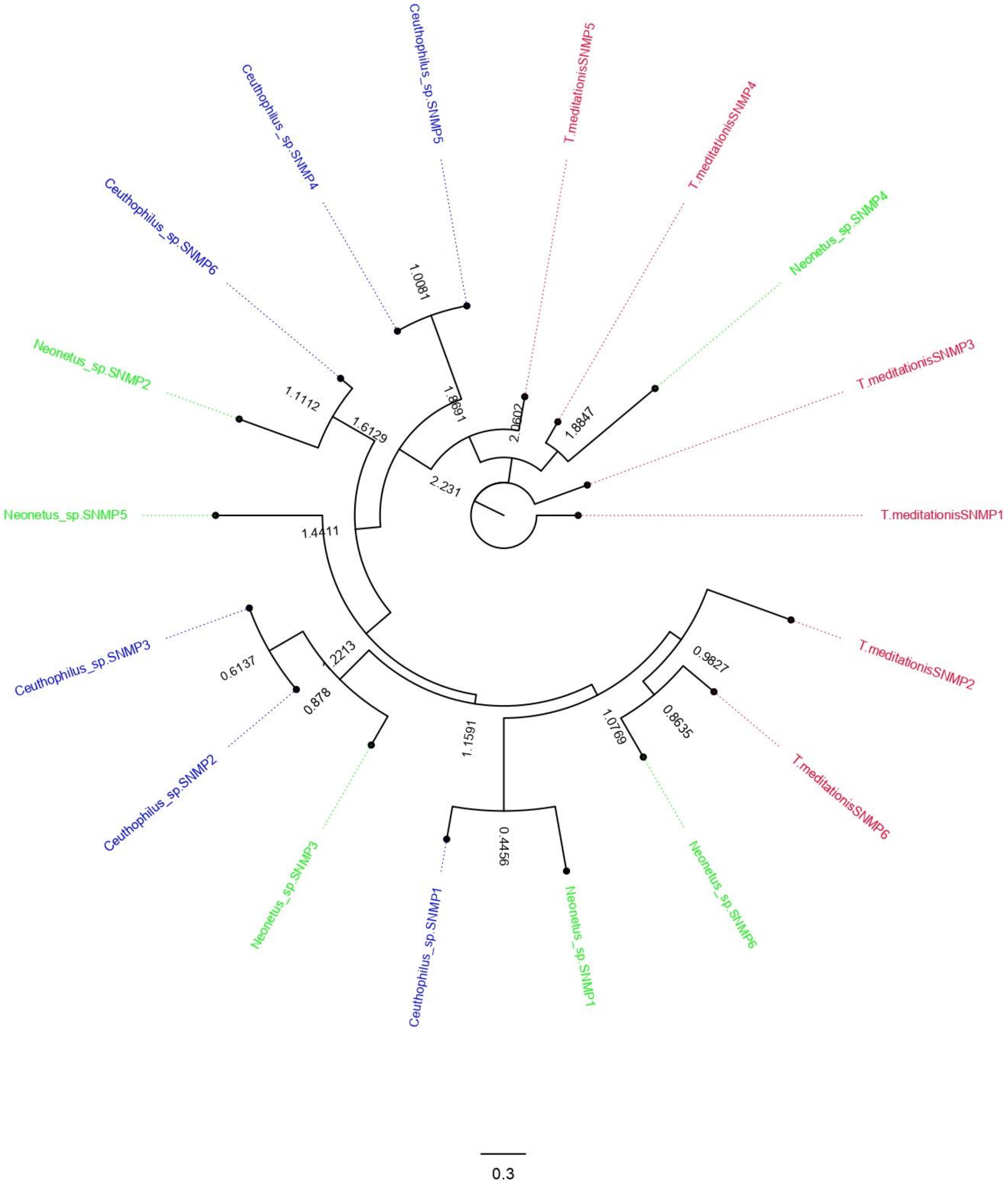

Figure S5. The ML phylogenetic tree of SNMP genes.

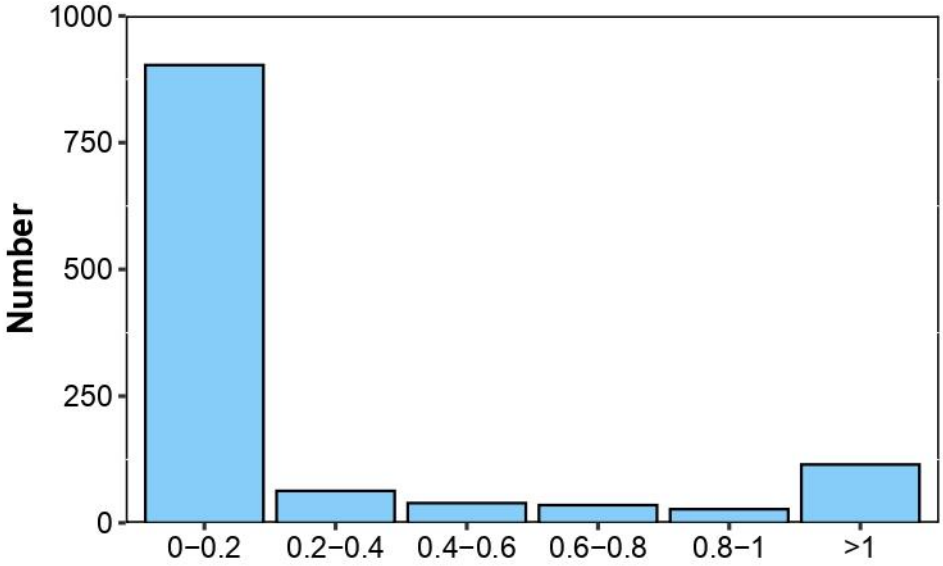

Figure S6. The distribution of branch-specific dN/dS of orthologous genes identified among *T.meditationis* and two camel crickets species.
